# Heat shock factor 1 protects germ cell proliferation during early ovarian differentiation in medaka

**DOI:** 10.1038/s41598-019-43472-4

**Published:** 2019-05-06

**Authors:** Fumiya Furukawa, Shin Hamasaki, Seiji Hara, Tomoya Uchimura, Eri Shiraishi, Natsumi Osafune, Hisanori Takagi, Takashi Yazawa, Yasuhiro Kamei, Takeshi Kitano

**Affiliations:** 1 Department of Biological Sciences, Graduate School of Science and Technology, Kumamoto University, Kumamoto, 860-8555 Japan; 2 Department of Biochemistry, Asahikawa Medical University, Asahikawa, Hokkaido, 078-8510 Japan; 3 Spectrography and Bioimaging Facility, National Institute for Basic Biology Core Research Facilities, National Institute for Basic Biology, Okazaki, 444–8585 Japan

**Keywords:** Reproductive biology, Animal physiology

## Abstract

The heat shock response is important for the viability of all living organisms. It involves the induction of heat shock proteins whose expression is mainly regulated by heat shock factor 1 (HSF1). Medaka (*Oryzias latipes*) is a teleost fish with an XX/XY sex determination system. High water temperature (HT) inhibits the female-type proliferation of germ cells and induces the masculinisation of XX medaka in some cases during gonadal sex differentiation. Here, we investigated the roles of HSF1 on the proliferation of germ cells using HSF1 knockout medaka. Loss of HSF1 function under HT completely inhibited the female-type proliferation of germ cells, induced the expression of the anti-Mullerian hormone receptor type 2 (*amhr2*) and apoptosis-related genes, and suppressed that of the dead end (*dnd*) and heat shock protein-related genes. Moreover, the loss of HSF1 and AMHR2 function under HT recovered female-type proliferation in germ cells, while loss of HSF1 function under HT induced gonadal somatic cell apoptosis during early sex differentiation. These results strongly suggest that HSF1 under the HT protects the female-type proliferation of germ cells by inhibiting *amhr2* expression in gonadal somatic cells. These findings provide new insights into the molecular mechanisms underlying environmental sex determination.

## Introduction

Organisms induce the expression of heat shock proteins (HSPs) when exposed to high temperature (HT). Under environmental stresses including HT, HSPs mainly act as molecular chaperones by assisting with protein folding, inhibiting protein denaturation, and maintaining protein homeostasis^1^. The expression of HSPs is mostly regulated by heat shock transcription factor (HSF). Vertebrate HSF consists of four families (HSF1–4), which are conserved from *Escherichia coli* to mammals. HSF1 has been reported to play an important role in the development of cancer, ageing, gametogenesis, and the response to thermal stress^2^. Studies on HSF1 knockout (KO) mice show that they survive into adulthood but with the disappearance of *hsp70* expression^2^. HSF1 KO male mice are fertile with normal testes^3^, while HSF1 KO female mice are infertile because of abnormal egg maturation^4^. Although HSF1 has been isolated in some poikilothermic vertebrates including rainbow trout^5^ and zebrafish^6^, its function in these animals has not been elucidated.

In poikilothermic vertebrates, sex determination is sometimes influenced by environmental factors such as temperature^7^, pH^8^, density^9^, and social factors^10,11^. Recently, it was reported that some HSPs show sexually dimorphic expression during gonadal sex differentiation in the American alligator with temperature-dependent sex determination (TSD)^12^. However, little is known about the roles of HSPs in environmental sex determination including TSD.

Medaka (*Oryzias latipes*) is a small laboratory fish with several desirable features, including a short generation time, small genome size, and the availability of several useful strains^13^. Transgenic techniques in medaka have proved useful^14^, and gene knockout systems using transcription activator-like effector nuclease (TALEN) or clustered regularly interspaced short palindromic repeats (CRISPR)/CRISPR-associated protein 9 have already been established^15,16^. Furthermore, DMY/dmrt1bY, the medaka sex-determining gene located on the Y chromosome, has been identified^17–19^. Thus, medaka is an excellent vertebrate model for the molecular genetic analysis of various biological phenomena including embryonic development and sex differentiation.

The first evidence of morphological sex differentiation in medaka is seen before hatching, occurring as a significant difference in the number of germ cells between the sexes: genetic females (XX) have more germ cells than genetic males (XY). These germ cells subsequently enter mitotic arrest in XY gonads, whereas they initiate oogenesis in XX gonads^20–22^. Previously, it was reported that HT (32–34 °C) during sex differentiation caused the masculinisation of XX medaka^23,24^, and led to the inhibition of germ cell proliferation and oocyte development in XX medaka^25,26^, suggesting that HT induces masculinisation of XX medaka by inhibiting the female-type proliferation of germ cells. However, the molecular mechanisms underlying the proliferation of germ cells are unclear. Here, we investigated the roles of HSF1 on germ cell proliferation during early sex differentiation using HSF1 knockout (KO) medaka established by TALEN. We also performed a comparative transcriptome analysis of HSF1 KO fish during gonadal sex differentiation using RNA sequencing (RNA-seq) technology, and analysed the involvement of candidate downstream genes on germ cell proliferation.

## Results

### Generation and phenotypic analysis of HSF1 KO medaka

**Figure 1:**
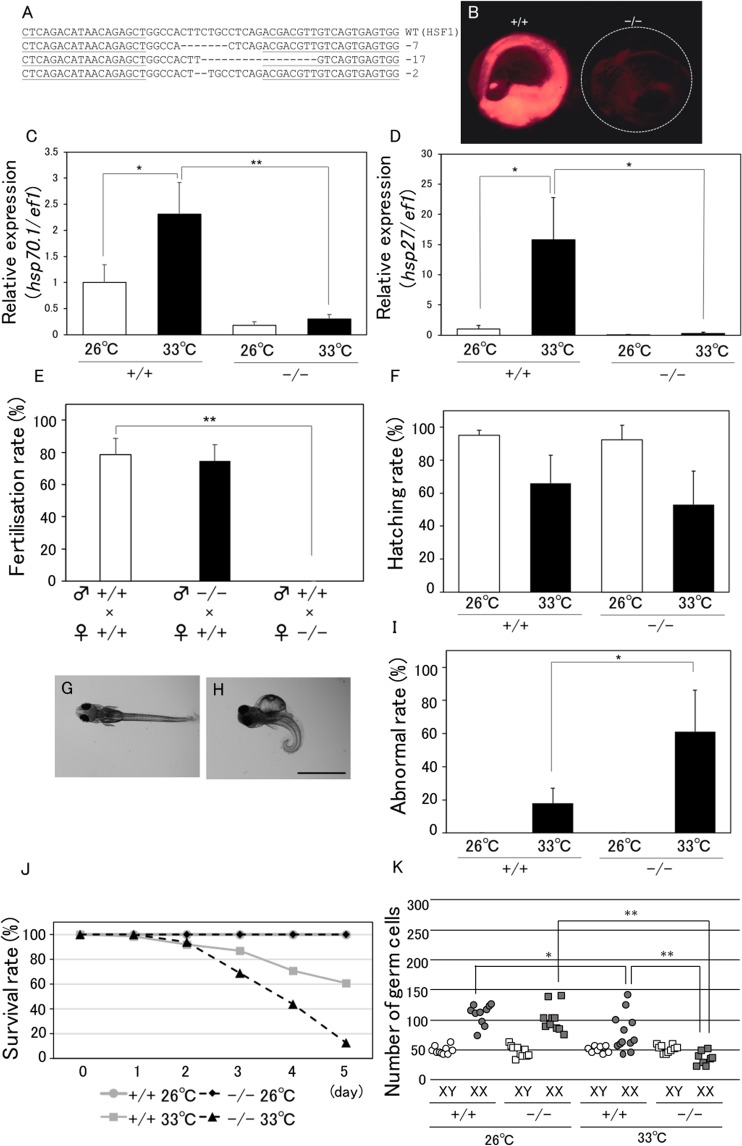
Phenotypes of HSF1 KO medaka. (**A**) Wild-type (WT) sequences of medaka *hsf1* and recognition sites of TALENs (underlined). The sizes of the deletions are shown to the right of each mutated sequence (−, deletions). (**B**) *hsp70*-DsRed Tg medaka enabling the monitoring of HSP70 expression by DsRed fluorescence. WT (+/+) and HSF1 homozygous mutated (–/–) embryos heated at 37 °C for 1 h were observed for DsRed fluorescence 1 day after heating. (**C**,**D**) *hsp70* (**C**) and *hsp27* (**D**) expression in whole-body fry at 0 dph. Relative expression was calculated based on the value of *ef1*. n = 4. (**E**) Fertilisation rate (fertilised eggs over obtained eggs) for 5 days in each of three mating groups: WT males and WT females, KO males and WT females, and WT males and KO females. n = 3. (**F**) Hatching rate (hatched fry over obtained embryos) in WT (+/+) and KO (–/–) embryos incubated at 26 °C or 33 °C. (**G**–I) Representative normal (**G**) and abnormal (**H**) fry incubated at 33 °C during 0 dpf to 0 dph, and the rate of abnormal fry (abnormal fry over hatched fry) (**I**). Scale bar, 1 mm. (**J**) Survival rate in WT (+/+) and KO (−/−) fry incubated at 26 °C or 33 °C during 0 dph to 5 dph. (**K**) Number of germ cells in WT (+/+) and KO (−/−) fry incubated at 26 °C or 33 °C during 0 dpf to 0 dph. *p < 0.05, **p < 0.01.

To generate HSF1 KO medaka by TALEN, *hsp70-DsRedExpress* transgenic (Tg) FLFII medaka were used^27,28^. This strain allows the visualisation of *hsp70*.*1* expression by DsRed fluorescence in living individuals^28^. Potential TALEN target sites were designed in exon 9 of the medaka *hsf1* gene, which contains essential phosphorylation sites required for the activation of HSF1^29^; it was expected that deletion of this region would result in a defective HSF1 function. Sequence analysis of TALEN-injected embryos revealed three types of *hsf1* sequence deletions (Fig. 1A). The injected fish were bred to adults, and 7-bp deletion mutants selected by genotyping were mated with wild-type (WT) Tg medaka. F1 generation heterogenous mutants were bred to adults and F2 generation HSF1 KO fish were produced by mating F1 mutants with each other. At and after the F3 generation, HSF1 KO medaka were produced by mating KO male fish with heterogenous female fish because KO female fish were infertile. After heating at 37 °C for 1 h, WT Tg medaka showed strong DsRed fluorescence (Fig. 1B, left), but this was not seen in HSF1 KO Tg fish before and after heating (Fig. 1B, right), and in WT Tg fish before heating (data not shown). Quantitative real-time PCR analysis showed that the expression levels of *hsp70* and *hsp27* were induced by 33 °C in WT, but not in HSF1 KO fish (Fig. 1C,D).

Next, we mated KO medaka with WT fish to investigate the fertility of KO fish. Fertilisation rates in the mating of WT females with KO males were similar to those observed for the mating of WT fish with each other. In comparison, fertilisation rates in the mating of WT males with KO females were 0%, indicating that KO males and females were fertile and infertile, respectively (Fig. 1E). To investigate the effects of HT (33 °C) in KO fish, we examined hatching, individual development, and survival rates in KO medaka produced by mating KO males with heterogenous females. Hatching rates in KO medaka were similar to those in WT fish (Fig. 1F), while significantly more abnormal KO fish incubated at 33 °C were seen than in WT fish incubated at 33 °C (Fig. 1G–I). Survival rates in KO medaka incubated at 26 °C were 100% over 5 days, similar to those in WT fish (Fig. 1J), whereas they were lower in KO fish incubated at 33 °C than in WT fish incubated at 33 °C (Fig. 1J).

To investigate the effects of HT on germ cell proliferation in HSF1 KO medaka during early sex differentiation, we counted the number of germ cells in WT and KO medaka incubated at 26 °C or 33 °C from 0 day post-fertilisation (dpf) to 0 day post-hatching (dph). Germ cell numbers in KO medaka incubated at 26 °C were similar to those in WT fish incubated at 26 °C (Fig. 1K). However, germ cell numbers in KO XY medaka incubated at 33 °C were similar to those in WT XY fish incubated at 33 °C (Fig. 1K), whereas germ cell number in KO XX fish incubated at 33 °C were significantly lower than those in WT XX fish incubated at 33 °C, suggesting that the effect of HT treatment was more pronounced in HSF1 KO XX medaka compared with WT XX fish.

### RNA-seq analysis of HSF1 KO XX medaka

**Figure 2:**
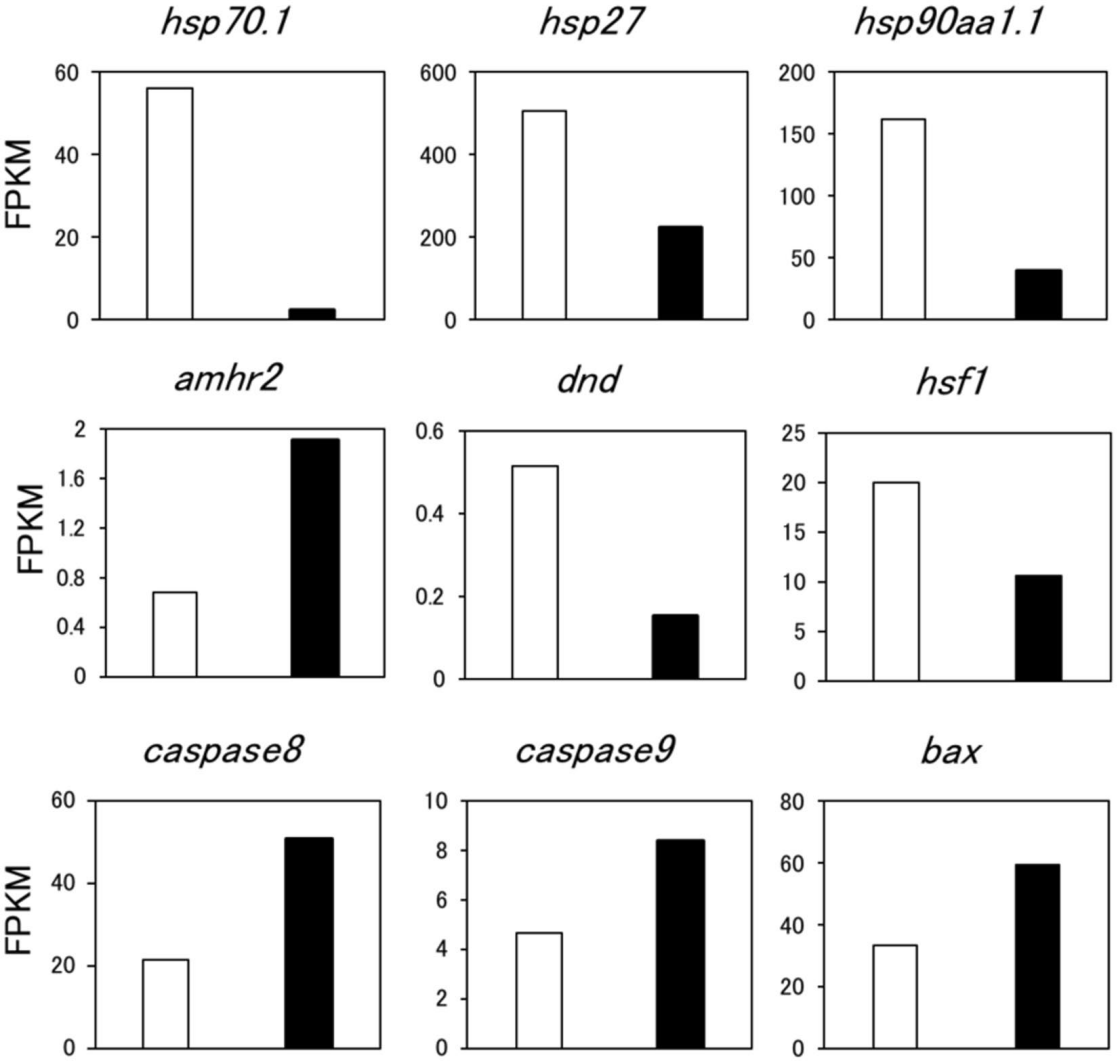
Genes with significant (p < 0.05) expression differences between HT-treated WT XX (open column) and HSF1 KO XX medaka (closed column) identified by RNA-seq analysis.

**Figure 3:**
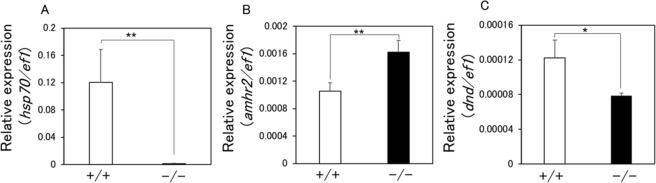
Real-time PCR analysis of HSF1 KO XX medaka. (**A**–**C**) *hsp70* (**A**), *amhr2* (**B**), and *dnd* (**C**) expression in the gonadal regions of HT-treated WT XX (+/+) and HSF1 KO XX (−/−) fry at 0 dph. Relative expression was calculated based on the value of *ef1*. **p < 0.01, n = 4.

To elucidate the molecular mechanisms underlying the decrease in germ cell number in HT-treated HSF1 KO XX medaka, we performed RNA-seq analysis using the gonadal regions of WT and HSF1 KO XX fish incubated at 33 °C. A total of 2454 genes showed expression differences between WT and KO fish. The expression levels (fragments per kilobase of transcript per million fragments mapped, FPKM) of *hsf1* and heat shock proteins (*hsp70*.*1*, *hsp27*, and *hsp90aa1*.*1*) were significantly decreased in KO fish compared with WT fish (Fig. 2, Supplementary Table S1). Moreover, expression of the anti-Mullerian hormone receptor type 2 (*amhr2*), which encodes an inhibitor of germ cell proliferation^30^, was increased in KO fish, while expression of the dead end (*dnd*), which encodes a germline-specific RNA-binding protein that regulates germ cell number^16^, was decreased in KO fish (Fig. 2). Furthermore, the expression of apoptosis-related genes (*caspase 8*, *caspase 9* and *bax*) was significantly increased in KO fish compared with WT fish, while expression of the internal control gene *ef1* and sex-specific genes (*foxl2*, *cyp19a1a*, *cyp19a1b*, *gsdf*, *amh* and *dmrt1*) showed no obvious differences between WT and KO XX fish (Fig. 2; Supplementary Table S1). Quantitative real-time PCR analysis confirmed that the expression of *hsp70*, *amhr2*, and *dnd* differed significantly between WT and KO XX fish (Fig. 3A–C).

### HT decreases germ cell number through AMHR2 function in HSF1 KO XX medaka

**Figure 4:**
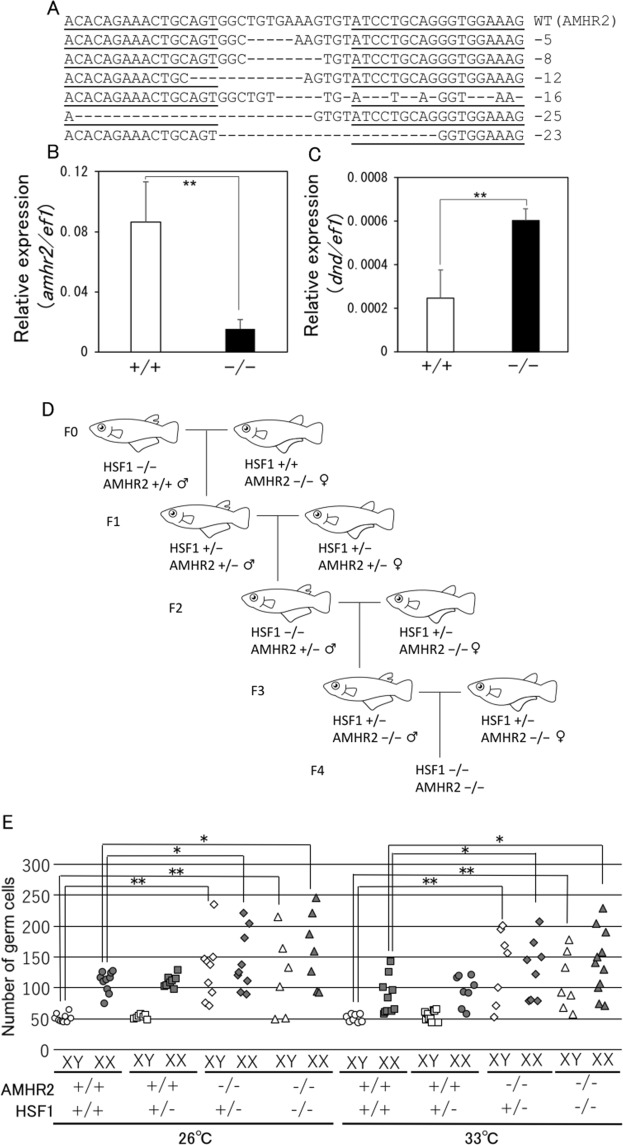
Phenotypic analysis of AMHR2 KO and HSF/AMHR2 KO medaka. (**A**) WT sequences of medaka *amhr2* and recognition sites of TALENs (underlined). Deletion sizes are shown to the right of each mutated sequence (−, deletions). (**B**,**C**) *amhr2* (**B**), and *dnd* (**C**) expression in gonadal regions of WT XX (+/+) and AMHR2 KO XX (−/−) fry at 0 dph. Relative expression was calculated based on the value of *ef1*. n = 5. (**D**) Mating scheme for HSF1/AMHR2 double KO medaka. (**E**) Number of germ cells in WT (+/+) and HSF1/AMHR2 KO (−/−) fry at 0 dph after HT treatment. *p < 0.05, **p < 0.01.

To confirm whether HSF1 protects germ cell proliferation through blocking AMHR2 function under HT, we generated HSF1 and AMHR2 double KO medaka. AMHR2 KO fish were first produced by TALEN using FLFII stock^27^. TALEN target sites were designed in exon 6 of medaka *amhr2*, which contains a kinase domain essential for the activation of AMHR2. Sequence analysis of TALEN-injected embryos revealed six types of deletion in the *amhr2* sequences (Fig. 4A). The injected fish were bred to adults, and 5-bp deletion mutants selected by genotyping were mated with WT FLFII medaka. F1 generation heterogenous mutants were bred to adults and F2 generation AMHR2 KO fish were produced by mating F1 mutants with each other. Quantitative real-time PCR analysis revealed that the expression levels of *amhr2* and *dnd* differed significantly between WT and AMHR2 KO XX fish (Fig. 4B,C). Next, HSF1 and AMHR2 double KO medaka were produced by mating AMHR2 KO female fish with HSF1 KO male fish (Fig. 4D). The germ cell number in AMHR2 KO XY and XX medaka incubated at 26 °C and 33 °C was significantly higher than in WT fish (Fig. 4E), similar to previous reports^30^. Additionally, the germ cell number in HSF1/AMHR2 KO XY and XX medaka incubated at 26 °C and 33 °C was significantly higher than in HSF1 KO (Fig. 1K) and WT fish (Fig. 4E), indicating that the decreased germ cell number in HSF1 KO XX medaka incubated at 33 °C is recoverable by loss of AMHR2 function.

### HT decreases the number of gonadal somatic cells by inducing apoptosis in HSF1 KO XX medaka

**Figure 5:**
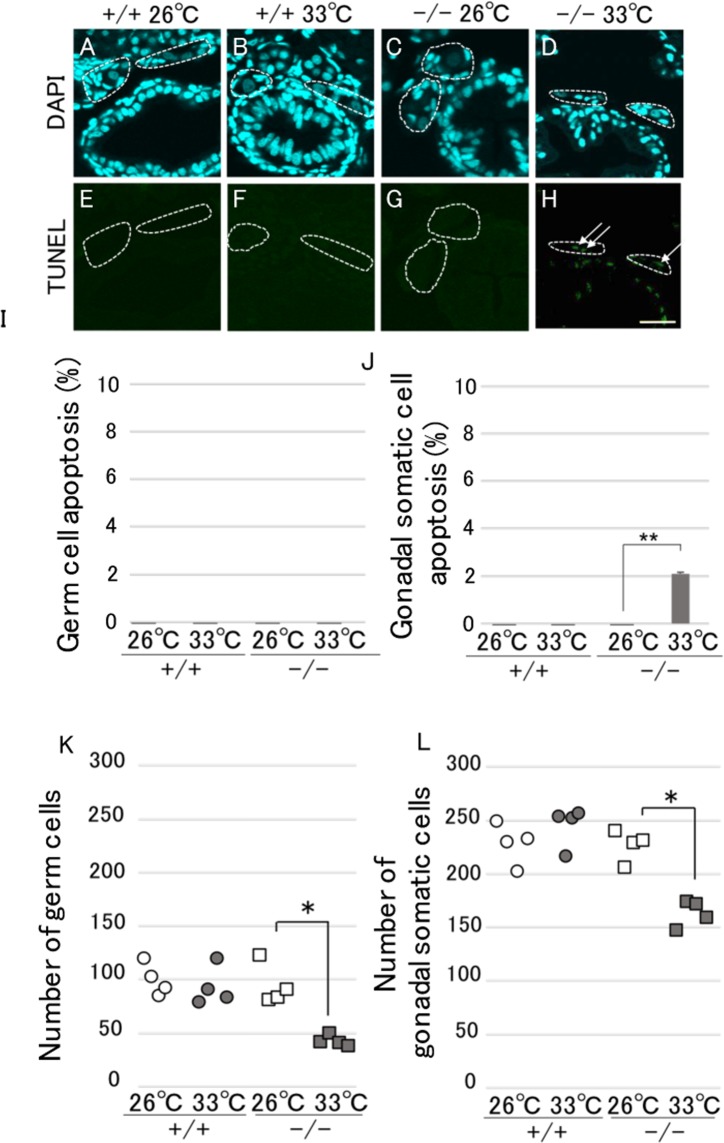
Detection of apoptosis in HSF1 KO XX medaka. (**A**–**H**) Apoptosis with TUNEL staining (green) and nuclear DAPI staining (blue) in gonadal regions of WT XX (**A**,**E**), HT-treated WT XX (**B**,**F**), HSF1 KO XX (**C**,**G**), and HT-treated HSF1 KO XX (**D**,**H**) fry. White dot, gonadal regions; white arrow, apoptosis in gonadal regions. Scale bar, 30 μm. n = 4. (**I**) Rate of apoptotic germ cells (TUNEL-positive germ cells over total germ cells). (**J**) Rate of apoptotic gonadal somatic cells (TUNEL-positive gonadal somatic cells over total gonadal somatic cells). (**K**,**L**) Number of germ cells (**K**) or gonadal somatic cells (**L**) of WT (+/+) and KO (−/−) fry at 0 dph. **p < 0.01.

RNA-seq analysis revealed the expressional induction of apoptosis-related genes in HSF1 KO XX medaka incubated at 33 °C compared with WT fish incubated at 33 °C. To confirm this, we used TUNEL staining as an indicator of apoptosis in WT and HSF1 KO fish. Apoptosis in germ cells was not detected in all individuals examined (Fig. 5A–I), while apoptosis in gonadal somatic cells was only detected in HSF1 KO XX fish incubated at 33 °C (Fig. 5H,J). However, the number of gonadal somatic cells in HSF1 KO XX fish incubated at 33 °C was significantly lower than in those incubated at 26 °C, similar to the findings of the germ cell number (Fig. 5K,L).

## Discussion

As a first step to elucidating the roles of HSPs on environmental sex determination including TSD, we successfully generated an HSF1 KO medaka line in which the expression of some *hsp* genes was not induced by HT, and performed phenotypic analysis. This KO fish showed normal development under normal temperatures; male adults had normal fertility, while female adults were infertile. This phenotype is similar to that seen in previous KO mouse studies, which reported decreased expression of *hsp* genes, normal fertility in KO males, but infertile KO females^2,3^. In mice, HSF1 KO females are infertile because HSF1 regulates oocyte meiosis through the expression of *hsp90a*^4^. Similarly, we observed reduced *hsp90a* expression in HSF1 KO medaka by RNA-seq analysis, indicating that this is also responsible for HSF1 KO female infertility. However, hatching rates in KO medaka were similar to those in WT fish, while more abnormal individuals and lower survival rates were seen in KO fish treated with HT than in WT fish treated with HT. A previous report using fish hepatocytes showed that an HSF1 is involved in the transcriptional induction of the *hsp70* gene^31^. Because HSF1 is typically activated under stress conditions including heat stress, HSP expression levels are also increased, which protects cells by repairing denatured proteins^1^. Therefore, the increased number of abnormal individuals and reduced survival rates in HSF1 KO fish may reflect the reduction in expression of some HSPs.

In medaka, the first appearance of morphological sex differentiation occurs before hatching as a significant difference in the number of germ cells between the sexes: XX medaka have more germ cells than XY fish^20–22^. It was also reported that HT inhibits the female-type proliferation of germ cells and induces the masculinisation of XX medaka during gonadal sex differentiation^23–26^. Our results showed that loss of HSF1 function under HT completely inhibited the female-type proliferation of germ cells, speculating the involvement of HSF1 in early ovarian differentiation in medaka. RNA-seq analysis in gonadal regions of 0-dph fry indicated that expression of the sex-specific genes (*foxl2*, *cyp19a1a*, *cyp19a1b*, *gsdf*, *amh* and *dmrt1*) had no obvious differences between WT and KO XX fish, suggesting that HSF1 does not regulate directly the expression of the sex-specific genes. On the other hand, RNA-seq and quantitative real-time PCR analyses revealed the induction of *amhr2* expression, which inhibits germ cell proliferation^30^, and the suppression of *dnd*, which regulates germ cell number^16^, in HSF1 KO fish treated with HT compared with WT fish treated with HT.

Previously, *amhr2* was shown to be specifically expressed in the gonadal somatic cells of both sexes of medaka^30^. Moreover, AMHR2 mutant medaka displayed phenotypic abnormalities including the excessive proliferation of germ cells and sex reversal in half of the homozygous XY fish. In this study, to investigate AMHR2 function in HSF1 KO medaka, we first generated AMHR2 KO fish using TALEN. Quantitative real-time PCR in the gonadal regions of 0-dph fry revealed the suppression of *amhr2* and induction of *dnd* in AMHR2 KO fish treated by HT compared with WT fish treated by HT. The germ cell number was significantly higher in HSF1/AMHR2 KO XX medaka treated by HT compared with HSF1 KO fish treated by HT, suggesting that *amhr2* expression decreases germ cell number in HSF1 KO XX medaka treated by HT. However, *dnd* is specifically expressed in the germ cells of both sexes and is a critical specifier of primordial germ cells in medaka^16,32,33^. Moreover, Dnd depletion by *dnd* knockdown specifically abolished PGCs, while its over-expression boosted PGCs^33^, suggesting that Dnd dosage controls PGC number in medaka. Therefore, loss of HSF1 function under HT appears to suppress *dnd* expression in the germ cells by inducing *amhr2* expression in the gonadal somatic cells.

In this study, RNA-seq analysis showed the induction of apoptosis-related genes in HSF1 KO XX medaka treated by HT compared with WT fish treated by HT. Accordingly, we detected apoptosis in WT and HSF1 KO fish using TUNEL staining. Apoptosis in germ cells was not detected in all individuals examined, while apoptosis in gonadal somatic cells was only detected in HSF1 KO XX fish treated by HT, followed by a decrease in the number of gonadal somatic cells in HSF1 KO XX fish treated by HT. A previous report using *hsp70-Venus* Tg medaka showed that the *hsp70*.*1* promoter, which is thought to be activated by HSF1, was functional under HT in many somatic cells (including gonadal somatic cells) except for germ cells of the fry^28^. Therefore, HSF1 under HT is likely to protect gonadal somatic cells rather than germ cells by elevating HSP expression during early sex differentiation in medaka. Recently, the treatment of mouse Sertoli cells with high concentrations of recombinant AMH elevated AMHRII expression, resulting in increased apoptosis^34^. Although the roles of AMH on gonadal somatic cells remain uncertain in medaka, the loss of HSF1 function under HT may inhibit the female-type proliferation of germ cells through increasing gonadal somatic cell apoptosis by excessive AMH signaling.

We have previously shown that HT inhibits the female-type proliferation of germ cells and induces masculinization of XX medaka by elevation of cortisol, the major glucocorticoid produced by the interrenal cells in teleosts^26^. In this study, HT inhibited the female-type proliferation of germ cells in WT and HSF1 KO XX medaka, but not in AMHR2 KO XX fish. Recently, cortisol treatment also inhibited the female-type proliferation of germ cells in WT XX medaka, but not in AMHR2 KO XX fish (Data not shown), suggesting that HT and cortisol treatment inhibit the germ cell proliferation through AMHR2 function in XX gonads during gonadal sex differentiation. Taken together, these results strongly suggest that HSF1 under the HT protects the female-type proliferation of germ cells by inhibiting *amhr2* expression against action of cortisol induced by HT.

In summary, we demonstrated that loss of HSF1 function under HT completely inhibited the female-type proliferation of germ cells, induced the expression of *amhr2* and apoptosis-related genes, and suppressed that of *dnd* and HSP-related genes, as shown by RNA-seq analysis. Moreover, the loss of HSF1 and AMHR2 function under HT recovered female-type proliferation in germ cells, while loss of HSF1 function under HT induced gonadal somatic cell apoptosis during early sex differentiation. These results strongly suggest that HSF1 under the HT protects the female-type proliferation of germ cells by inhibiting *amhr2* expression in gonadal somatic cells. These findings provide new insights into the molecular mechanisms underlying environmental sex determination including TSD.

## Methods

### Ethics statement

The study was performed using protocols approved by the Animal Care and Use Committee of Kumamoto University (Approval number: A28-029). All experiments were performed in accordance with relevant guidelines and regulations.

### Animals

The FLFII medaka stock was used^27^, which allows the identification of genotypic sex by the appearance of leucophores at 2 dpf, before the onset of sex differentiation. The *hsp70-DsRedExpress* transgenic medaka strain was generated by injecting the *pDsRed-Express2-1* vector (Clontech, Palo Alto, CA) fused to the regulatory region of medaka *hsp70*.*1*^28^ into fertilised eggs of FLFII stock. All injected embryos were bred to adults and only F1 embryos possessing DsRed fluorescence were selected and used to produce succeeding generations. Fish embryos and fry were maintained in ERM (17 mM NaCl, 0.4 mM KCl, 0.27 mM CaCl_2_.2H_2_O, 0.66 mM MgSO4, pH 7) at 26 °C under a 14-h light and 10-h dark cycle. Fry images were captured using a BZ-9000 BioRevo fluorescence microscope (Keyence Co., Osaka, Japan).

### HT treatment

HT experiments were carried out by incubating medaka embryos in ERM at 33 °C from 0 dpf to 0 dph.

### Design and construction of TALENs

Potential TALEN target sites in the *hsf1* or *amhr2* locus were identified using the TALEN Targeter program (https://tale-nt.cac.cornell.edu/node/add/talen-old)^35^. TAL repeats were assembled by the Golden Gate assembly method^36^ with slight modifications^37^. The fragments were purified using the Wizard SV Gel and PCR clean-up system (Promega, Madison, WI), and then cloned into TALEN expression vectors containing the SP6 promoter and either the DD or RR heterodimeric FokI domain.

### RNA preparation and microinjection

Expression vectors for TALENs were linearised by digestion and capped RNAs were synthesised using the mMESSAGE mMACHINE SP6 Kit (Life Technologies, Gaithersburg, MD). Pairs of RNA for the TALENs were injected into one-cell embryos using Nanoject II (Drummond Scientific Co., Broomall, PA).

### Genotyping

Genomic DNA was extracted from medaka embryos as previously described^26^. PCR was carried out using AmpliTaq Gold (Applied Biosystems, Foster, CA) with the following primers: forward primer for *hsf1*, 5′-ATGGCACTCCTGATGCTCAG-3ʹ; forward primer for *amhr2*, 5ʹ-ACAGGTTGTGGGACAAGGAC-3′; reverse primer for *hsf1*, 5ʹ-GACAACGTCGTCTGAGGCAG-3ʹ; reverse primer for *amhr2*, 5ʹ-GAGCAATCCCAGCATGCCTC -3ʹ. PCR conditions were as follows: preheating at 95 °C for 10 min, then 45 cycles of 94 °C for 30 s, 59 °C for 30 s, and 72 °C for 30 s, and a final extension at 72 °C for 5 min. PCR products were subcloned into the pT7Blue vector (Novagen, Madison, WI) and sequenced with the GenomeLabTM GeXP Genetic Analysis System (Beckman Coulter, Fullerton, CA).

### Cell counts

Fry fish at 0 dph were fixed in Bouin’s solution at 4 °C overnight, embedded in paraffin, sectioned serially at a thickness of 5 μm, and stained with haematoxylin and eosin as previously described^16^. Germ cells were counted for each fish using a MZFLIII microscope (Leica Microsystems, Wetzlar, Germany).

### RNA-seq analysis

Total RNA was extracted from the gonadal regions (20 pooled fishes) of medaka fry at 0 dph using NucleoSpin RNA XS (Takara Bio Inc., Shiga, Japan). The RNA integrity was assessed by the Agilent 2100 Bioanalyzer system (Agilent Technologies, Santa Clara, CA). mRNA sequencing libraries for RNA-seq were constructed with the TruSeq RNA Library Prep kit v2 (Illumina, San Diego, CA) and then paired-end (2 × 100 bp) sequencing was performed using the Illumina HiSeq. 4000 Sequencing System (Illumina) at the Beijing Genomics Institute.

### RNA-seq data processing

Raw paired-end reads were subjected to quality control, and clean reads were filtered with FASTX-Toolkit (http://hannonlab.cshl.edu/fastx_toolkit/index.html). The read quality was checked with FastQC 0.11.3 (http://www.bioinformatics.bbsrc.ac.uk/projects/fastqc/). Clean reads were mapped to the Hd-rR medaka assembly in the Ensembl database using Tophat 2.1.0 software^38^. Gene expression levels were quantified by Cufflinks 2.2.1 (http://coletrapnell-lab.github.io/cufflinks/) and normalised by the FPKM method.

### Real-time PCR

Total RNA was extracted from medaka fry at 0 dph using ISOGEN (Nippon Gene, Tokyo, Japan) as previously described^39^. One µg of RNA was then reverse transcribed using an RNA PCR kit (Applied Biosystems) at 42 °C for 30 min. Quantitative real-time PCR was performed using SYBR Green I Master Mix (Roche, Mannheim, Germany) on a LightCycler 480 (Roche) with the following primers: forward primer for *hsp70*, 5′-AAGCCGAGGACGAGCAGCA-3′ and reverse primer for *hsp70*, 5′-TTGCAGACCTTCTCCAGCTC-3′; forward primer for *hsp27*, 5′-CGCTACTCTTACAGACTCCA-3′ and reverse primer for *hsp27*, 5′-GATGTATTGGGAGTCTGGAC-3′; forward primer for *amhr2*, 5′-GCAGGTTGTGGGACAAGGAC-3′ and reverse primer for *amhr2*, 5′-GAGCAATCCCAGCATGCCTC-3′; forward primer for *dnd*, 5′-AGGTGGTGAACTTGGAGCGG-3′ and reverse primer for *dnd*, 5′-CTGCAGCAGCTCCTCCTGC-3′; and forward primer for *ef1*, 5′-TGAGATGGGCAAGGGCTCCT-3′ and reverse primer for *ef1*, 5′-GCTGGGTTGTAGCCGATCTT-3′. PCR conditions were as follows: preheating at 95 °C for 5 min, then 45 cycles of 95 °C for 10 s, 59 °C for 10 s, and 72 °C for 10 s. The copy number values of *hsp70* and *hsp27* were normalised to that of *ef1*.

### Detection of apoptosis

Fry fish at 0 dph were fixed in 4% paraformaldehyde/PBS at 4 °C overnight, embedded in paraffin, and sectioned serially at 10 μm thickness. They were then stained with the Apoptotic/Necrotic Cell Detection kit (Takara Bio Inc.) and mounted with Vectashield HardSet mounting medium with DAPI H-1500 (Vector Laboratories Inc., Burlingame, CA). All germ cells and gonadal somatic cells were counted based on DAPI staining under a Fluoview FV10i confocal microscope (Olympus, Tokyo, Japan).

### Statistical analysis

Experimental results were tested using Levene’s test for homogeneity of variance. Data were analysed by the Student’s t-test or by one-way ANOVA followed by the Turkey’s multiple comparison test using SPSS statistics 20 (IBM Corp., Armonk, NY).

## Supplementary information

Supplementary Table S1

## References

[1] Young JC, Agashe VR, Siegers K, Hartl FU (2004). Pathways of chaperone-mediated protein folding in the cytosol. Nat. Rev. Mol. Cell Biol..

[2] Xiao X (1999). HSF1 is required for extra-embryonic development, postnatal growth and protection during inflammatory responses in mice. EMBO J..

[3] Wang G (2004). Essential requirement for both hsf1 and hsf2 transcriptional activity in spermatogenesis and male fertility. Genesis.

[4] Metchat A (2009). Mammalian heat shock factor 1 is essential for oocyte meiosis and directly regulates Hsp90alpha expression. J. Biol. Chem..

[5] Le Goff P (2004). Intracellular trafficking of heat shock factor 2. Exp. Cell Res..

[6] Råbergh CM (2000). Tissue-specific expression of zebrafish (*Danio rerio*) heat shock factor 1 mRNAs in response to heat stress. J. Exp. Biol..

[7] Baroiller JF, Guiguen Y, Fostier A (1999). Endocrine and environmental aspects of sex differentiation in fish. Cell. Mol. Life Sci..

[8] Rubin DA (1985). Effect of pH on sex ratio in cichlids and a poeciliid (Teleostei). Copeia.

[9] Tabata K (1995). Reduction of female proportion in lower growing fish separated from normal and feminized seedlings of hirame *Paralichthys olivaceus*. Fish. Sci..

[10] Francis RC (1984). The effects of bidirectional selection for social dominance on agonistic behavior and sex ratios in the paradise fish (*Macropodus Opercularis*). Behaviour.

[11] Francis RC, Barlow GW (1993). Social control of primary sex differentiation in the Midas cichlid. Proc. Natl. Acad. Sci. USA.

[12] Kohno S (2010). Potential contributions of heat shock proteins to temperature-dependent sex determination in the American alligator. Sex. Dev..

[13] Ishikawa Y (2000). Medakafish as a model system for vertebrate developmental genetics. BioEssays.

[14] Ozato K (1986). Production of transgenic fish: introduction and expression of chicken delta-crystallin gene in medaka embryos. Cell Differ..

[15] Ansai S (2013). Efficient targeted mutagenesis in medaka using custom-designed transcription activator-like effector nucleases. Genetics.

[16] Sawamura R, Osafune N, Murakami T, Furukawa F, Kitano T (2017). Generation of biallelic F0 mutants in medaka using the CRISPR/Cas9 system. Gene. Cell..

[17] Matsuda M (2002). DMY is a Y-specific DM-domain gene required for male development in the medaka fish. Nature.

[18] Matsuda M (2007). DMY gene induces male development in genetically female (XX) medaka fish. Proc. Natl. Acad. Sci. USA.

[19] Nanda I (2002). A duplicated copy of DMRT1 in the sex-determining region of the Y chromosome of the medaka, *Oryzias latipes*. Proc. Natl. Acad. Sci. USA.

[20] Satoh N, Egami N (1972). Sex differentiation of germ cells in the teleost, *Oryzias latipes*, during normal embryonic development. J. Embryo. Exp. Morph..

[21] Hamaguchi S (1982). A light- and electron-microscopic study on the migration of primordial germ cells in the teleost, *Oryzias latipes*. Cell. Tissue. Res..

[22] Kobayashi T (2004). Two DM domain genes, DMY DMRT1, involved in testicular differentiation and development in the medaka, *Oryzias latipes*. Dev. Dyn..

[23] Sato T (2005). Induction of female-to-male sex reversal by high temperature treatment in medaka, *Oryzias latipes*. Zoolog. Sci..

[24] Hattori RS (2007). Temperature-dependent sex determination in Hd-rR medaka *Oryzias latipes*: Gender sensitivity, thermal threshold, critical period, and DMRT1 expression profile. Sex. Dev..

[25] Selim KM (2009). Effects of high temperature on sex differentiation and germ cell population in medaka, *Oryzias latipes*. Aquaculture.

[26] Hayashi Y (2010). High temperature causes masculinization of genetically female medaka by elevation of cortisol. Mol. Reprod. Dev..

[27] Wakamatsu Y (2003). Establishment of new medaka (*Oryzias latipes*) stocks carrying genotypic sex markers. Environ. Sci..

[28] Oda S (2010). Identification of a functional medaka heat shock promoter and characterization of its ability to induce exogenous gene expression in medaka in vitro and in vivo. Zoolog. Sci..

[29] Holmberg CI (2001). Phosphorylation of serine 230 promotes inducible transcriptional activity of heat shock factor 1. EMBO J..

[30] Morinaga C (2007). The hotei mutation of medaka in the anti-Mullerian hormone receptor causes the dysregulation of germ cell and sexual development. Proc. Natl. Acad. Sci. USA.

[31] Airaksinen S, Råbergh CM, Sistonen L, Nikinmaa. M (1998). Effects of heat shock and hypoxia on protein synthesis in rainbow trout (*Oncorhynchus mykiss*) cells. J. Exp. Biol..

[32] Liu L (2009). Medaka dead end encodes a cytoplasmic protein and identifies embryonic and adult germ cells. Gene Expr. Patterns.

[33] Hong N (2016). Dnd is a critical specifier of primordial germ cells in the medaka fish. Stem Cell Rep..

[34] Rehman ZU (2017). Role and mechanism of AMH in the regulation of Sertoli cells in mice. J. Steroid Biochem. Mol. Biol..

[35] Doyle EL (2012). TAL Effector-Nucleotide Targeter (TALE-NT) 2.0: tools for TAL effector design and target prediction. Nucleic Acids Res..

[36] Cermak T (2011). Efficient design and assembly of custom TALEN and other TAL effector-based constructs for DNA targeting. Nucleic Acids Res..

[37] Sakuma T (2013). Efficient TALEN construction and evaluation methods for human cell and animal applications. Gene. Cell..

[38] Andrews S, Trapnell C, Pachter L, Salzberg SL (2009). TopHat: discovering splice junctions with RNA-Seq. Bioinformatics.

[39] Kitano T, Hayashi Y, Shiraishi E, Kamei Y (2012). Estrogen rescues masculinization of genetically female medaka by exposure to cortisol or high temperature. Mol. Reprod. Dev..

