# Supplementary material for: Heat shock factor 1 protects germ cell proliferation during early ovarian differentiation in medaka
Source: Sci Rep. 2019 May 6;9:6927. doi: 10.1038/s41598-019-43472-4 (PMC6502851; doi:10.1038/s41598-019-43472-4)
Supplement: Supplementary file 1 — Supplementary Table S1 [file 41598_2019_43472_MOESM1_ESM.pdf]

Heat shock factor 1 protects germ cell proliferation during early ovarian differentiation in medaka

Fumiya Furukawa[1], Shin Hamasaki[1], Seiji Hara[1], Tomoya Uchimura[1], Eri Shiraishi[1], Natsumi Osafune[1], Hisanori Takagi[1], Takashi Yazawa[2], Yasuhiro Kamei[3] & Takeshi Kitano[1]

[1]Department of Biological Sciences, Graduate School of Science and Technology, Kumamoto University, Kumamoto 860-8555, Japan. [2]Department of Biochemistry, Asahikawa Medical University, Asahikawa, Hokkaido 078-8510, Japan. [3]Spectrography and Bioimaging Facility, National Institute for Basic Biology Core Research Facilities, National Institute for Basic Biology, Okazaki 444–8585, Japan. Correspondence and requests for materials should be addressed to T.K. (email: tkitano@kumamoto-u.ac.jp)

Supplementary Table S1. Genes examined by RNA-seq analysis. Value log2 (fold-change) represents the logarithm of FPKM.

Gene	FPKM		log2(fold change)
	WT	HSF1 KO	
hsf1	19.9736	10.7239	-0.897268
hsf2	10.1859	5.88019	-0.792643
hsf2bp	1.71990	1.45330	-0.242992
hsf4	0.0614395	0.0566860	-0.116174
hsp70.1	56.4562	2.54311	-4.47247
hsp90aa1.1	163.257	40.0666	-2.02667
hsp90aa1.2	31.1258	16.4092	-0.923608
hsp90ab1	2341.12	2081.86	-0.169329
hsp90ab1	1032.48	933.118	-0.145978
hsp90b1	283.355	363.5365	0.359490
hspa12a	4.95635	2.67977	-0.887172
hspa12b	1.49872	0.775428	-0.950667
hspa13	7.27108	9.80543	0.431410
hspa14	15.2382	19.7159	0.371672
hspa4a	20.9678	10.6189	-0.981541
hspa4b	84.59250	76.96310	-0.13636
hspa4l	4.78437	4.08554	-0.227802
hspa5	324.034	394.317	0.283209
hspa8	50.4852	36.3321	-0.474616
hspb1	508.726	224.961	-1.17722
hspb11	110.193	466.370	2.08145
hspb15	41.6360	43.2955	0.0563857
hspb8	58.3947	187.963	1.68654
hspbap1	5.17284	7.33334	0.503514
hspbp1	25.2838	25.4804	0.0111775
hspd1	7.90706	5.96997	-0.405419
hspd1	123.841	162.144	0.388788

hspe1	375.510	555.975	0.566167
hspg2	7.97305	6.72602	-0.245379
foxl2	0.179343	0.206517	0.203543
cyp19a1a	0.0631787	0.0865760	0.454530
cyp19a1b	0.512715	0.576616	0.169455
gsdf	4.92977	4.05337	-0.282398
amh	3.15737	2.75156	-0.198472
amhr2	0.67471	1.89983	1.493540
dmrt1	0	0	
dnd	0.51471	0.150731	-1.771770
bax	33.6838	59.6666	0.824869
caspase8	21.52380	50.5877	1.232850
caspase9	4.62048	8.33648	0.851397
ef1	3594.24	3437.81	-0.0641978
